# Entrepreneurial Passion and Personality: The Case of Academic Entrepreneurship

**DOI:** 10.3389/fpsyg.2018.02697

**Published:** 2019-01-09

**Authors:** Martin Obschonka, Julia Moeller, Maximilian Goethner

**Affiliations:** ^1^Australian Centre for Entrepreneurship Research, Queensland University of Technology, Brisbane, QLD, Australia; ^2^Leipzig University, Leipzig, Germany; ^3^Friedrich Schiller University Jena, Jena, Germany

**Keywords:** entrepreneurial passion, personality, profiles, entrepreneurial behavior, mediation, rational choice, social learning theory, social identity theory

## Abstract

Since entrepreneurial thinking and acting within organizations is increasingly important for the success of organizations, entrepreneurial passion is an emerging key construct in the study of organizational behavior. Here we quantify effects of personality traits on entrepreneurial passion in organizations, thereby comparing a person- vs. variable-oriented trait approach and testing such effects against alternative explanation models (rational choice approach, social learning approach, and social identity approach). Analyzing data from *N* = 137 German scientists across two measurement occasions, structural equation modeling revealed that an entrepreneurial Big Five profile (person-oriented approach), but none of the single Big Five traits (variable-oriented approach), predicted entrepreneurial passion (which in turn mediated the link between this domain-specific personality profile and entrepreneurial behavior). Likewise, the entrepreneurial personality profile, but not the single Big Five traits, predicted the simultaneous occurrence of entrepreneurial passion and behavior (passionate entrepreneurial behavior). Interestingly, the alternative explanation models (rational choice approach, social learning approach, and social identity approach) failed to predict entrepreneurial passion and passionate entrepreneurial behavior. The results suggest that the basic entrepreneurial personality character of a person contributes to the shaping of his or her entrepreneurial passion, which is relevant for actual entrepreneurial activity. The results thus illustrate how a person-oriented trait approach can inform the study, and concepts of, entrepreneurial passion.

## Introduction

Many experts agree that passion is “at the heart” of entrepreneurship (Baum and Locke, 2004; Cardon et al., 2005, 2009a,b; Cardon, 2008; Klaukien and Patzelt, 2008; Chen et al., 2009; Klaukien and Breugst, 2009) because thinking and acting entrepreneurially arguably requires a strong passion that fuels the personal agency, proactivity, creativity, risk-taking, aspiration, resilience, and persistence that are needed in entrepreneurship (Cardon et al., 2009b). Moreover, entrepreneurial passion seems to be important in social interactions, because entrepreneurs who display passion are perceived as potentially more successful by investors, clients, and employees (e.g., Baum and Locke, 2004; but see also Chen et al., 2009; Cardon and Kirk, 2015). Successful entrepreneurs often describe a strong passion for entrepreneurship as one of their most important success factors (Isaacson, 2011).

Interestingly, individuals showing entrepreneurial activity often differ in their intensity of entrepreneurial passion (e.g., not every entrepreneur is passionate about his or her entrepreneurial activity). However, we still know very little about the origins and predictors of entrepreneurial passion. If passion is indeed that important for entrepreneurial activity, it is crucial to know more about the antecedents of interindividual differences in entrepreneurial passion, for example, to inform intervention programs and policy measures aimed at promoting not only entrepreneurial thinking and acting, which has become a major aim on the political agenda (Audretsch, 2007), but also *passionate* entrepreneurship (Cardon et al., 2009b). The central aim of this study was thus to provide new insights into *the antecedents* of entrepreneurial passion, and thus of entrepreneurial behavior. We put a special focus on the role of personality traits and structure (Obschonka and Stuetzer, 2017)—thereby testing the general assumption whether an entrepreneurial personality structure (e.g., entrepreneurial constellation of basic, relatively stable traits) makes passionate entrepreneurship more likely. Or in other words, we essentially test whether interindividual differences in an “entrepreneurial character” predict interindividual differences in passionate entrepreneurial activity.

The present study thus contributes, first, to entrepreneurship research in that it offers new insights into the antecedents of entrepreneurial passion. Second, it also contributes to the specific field of *academic* entrepreneurship (Shane, 2004). Finally, such research on entrepreneurial passion also makes contributions to research on the general role of passion in the work context, which is a growing field in organizational research and vocational behavior (Vallerand and Houlfort, 2003; Perrewé et al., 2014; Egan et al., 2017). For example, research indicates that passion plays a vital role for work motivation and work outcomes in general (Carbonneau et al., 2008; Burke and Fiksenbaum, 2009), in work-related stress processes (Vallerand et al., 2010), in job transitions (Houlfort et al., 2015; Huyghe et al., 2016), in job creativity (Liu et al., 2011), in leadership (Egan et al., 2017), and in feeling a “calling” for one's vocation (Duffy and Dik, 2013). However, despite this research, Perrewé et al. (2014) recently concluded that “in the workplace, passion is a highly sought-after, yet poorly understood (and cultivated), worker attribute” (p. 145). Particularly unclear is why some people develop a certain passion at work while others do not.

## Entrepreneurial Passion in Academia as Domain-Specific Passion

In the present study, we focus on entrepreneurial passion and behavior in the organizational context of academia (scientists working in research institutions). This has three main reasons. First, the majority of entrepreneurs engage in enterprising activity after a period of employment in established organizations (Nanda and Sørensen, 2010). Hence, it is essential to know more about the development of entrepreneurial mindsets within established organizations (Hisrich et al., 2007). Second, academic entrepreneurship—the commercialization of science through entrepreneurial behavior of scientists (Shane, 2004; Huyghe et al., 2016)—has become a particularly relevant topic on today's political agenda seeking to promote innovation and competitive advantage by fostering the exploitation, application, and commercialization of new scientific knowledge (Audretsch, 2007; Perkmann et al., 2013). New scientific knowledge is often tacit and therefore person-embodied. It requires the active involvement of the knowledge-generating scientist when transforming new knowledge into entrepreneurial ideas, products, or services (Shane, 2004). Hence, the individual scientist acting entrepreneurially has become a new research focus in recent years (Aldridge and Audretsch, 2011). Entrepreneurial passion in scientists, in turn, is deemed a highly relevant psychological aspect behind academic entrepreneurship (Huyghe et al., 2016). Knowing more about its antecedents can contribute to a better understanding of the motivational drivers of academic entrepreneurship. Third, scientists' entrepreneurial passion can be understood as domain-specific passion. While passion researchers also view general passion (e.g., work passion in general) as highly relevant for individual behavior and achievement (Vallerand et al., 2003; Perrewé et al., 2014). Entrepreneurial universities and scientists are hot topics on the political agenda interested in boosting innovation and technology transfer (Audretsch, 2007; Aldridge and Audretsch, 2011). Entrepreneurial passion in scientists is deemed a highly relevant psychological aspect of academic entrepreneurship (Huyghe et al., 2016).

Research on predictors of domain-specific passion is still relatively scarce. On one hand, some studies emphasize the role of malleable aspects, such as training and effort as drivers of entrepreneurial passion (Gielnik et al., 2015, 2017) and the role of fluctuating situational determinants on passion for diverse activities (Moeller et al., 2017). On the other hand, other approaches suggest that relatively stable personality characteristics predict passion for diverse activities in general (Wang and Yang, 2008; Tosun and Lajunen, 2009; Balon et al., 2013) and entrepreneurial passion in particular (Barrick and Mount, 1991; Judge and Ilies, 2002; Duckworth et al., 2007; Cardon et al., 2009b). Existing research also focused on the effect of entrepreneurial identity aspects on entrepreneurial passion, and found that passion was predicted by identity centrality, but not identity salience (Murnieks et al., 2014). In the present study, we follow the perspective giving relatively stable personality characteristics a unique role behind passionate entrepreneurship.

## The Personality-Approach to Passion

Passion researchers have called for more research on the role of dispositions as drivers of passion. Perrewé et al. (2014), for example, explicitly suggest that “as a starting point, research should focus on the dispositional and physiological underpinnings of the passion construct” (p. 147). This directs attention toward a personality-based approach to passion.

Psychological research established the central role of personality in determining human motivation and behavior, thereby underscoring the “power of personality” (Roberts et al., 2007). The focus on inter-individual personality differences can be regarded as a classic approach in work psychology (Fouad, 2007) and also in psychological entrepreneurship research (Hisrich et al., 2007; Obschonka and Fisch, 2018). Here, research established that personality characteristics such as the Big Five traits are a functional part of the entrepreneurial mindset (Obschonka and Stuetzer, 2017) but to our knowledge, no study exists linking these personality traits to entrepreneurial passion.

Some psychologists describe passion as some sort of a stable disposition of a person to engage and persist in particular types of activities, which underscores the relevance of looking at passions for *specific* work behaviors/tasks (Vallerand et al., 2003; Collewaert et al., 2016; Moeller et al., 2017). For example, psychological passion studies showed that about 20% of the variance in momentary experiences of passion is due to person-specific determinants and that the individual likelihood to have many vs. few passionate experiences in everyday life situations remains relatively stable across a period of years (Moeller et al., 2017). These considerations and findings thus hint at the relevance of relatively stable dispositions such as personality traits in determining passion.

Only a few studies (using cross-sectional designs) have addressed the relationship between passion and personality, most of which have been conducted outside work contexts. First evidence for cross-sectional links between passion (regarding Vallerand et al.'s definition) and the Big Five personality traits was reported by Wang and Yang (2008), who found that openness to experience, agreeableness, and conscientiousness were related to passion for online shopping activities. These findings were corroborated by Balon et al. (2013), who found that harmonious passion for people's favorite activities was positively correlated with their conscientiousness, extraversion, agreeableness, and openness, whereas obsessive passion for a person's favorite activities was negatively related to agreeableness (all effect sizes were small). Moreover, in their study on links between Eysenckian personality traits and passion for internet activities, Tosun and Lajunen (2009) found that psychoticism was positively related to both harmonious and obsessive passion, whereas extraversion was related to harmonious passion and neuroticism was unrelated to passion for internet activities. Examining more specific personality facets has revealed stronger correlations to passion than the abstract Big Five personality factors: A number of studies found passion correlated with reward and emotion-related extraversion facets, including sensitivity to reward, high arousal positive affectivity, trait cheerfulness and different aspects of dependency (Moeller et al., 2015). In contrast, passion was unrelated to the extraversion facet sensation seeking in different samples. These results were replicated in several domains (including soccer, poker play, dance and martial arts), age groups (adolescents to middle-aged adults), and countries (Germany and Brazil). Other specific personality dispositions and their link to passion were studied by Vallerand et al. (2006), who assumed that the disposition to act more or less autonomously influenced the type of experienced passion. The authors found that, as predicted by the dual model of passion (harmonious vs. obsessive passion, Vallerand et al., 2003), autonomous personality orientation correlated with harmonious passion and controlled personality orientation correlated with obsessive passion.

In sum, passion seems to be influenced by relatively stable personality traits, but the previous findings on the relationship between personality and passion are inconsistent across studies. Much of the previous research has focused on the aspects of passion and personality that are invariant across contexts. But is a passion for a voluntary recreational leisure activity such as collecting stamps or computer gaming comparable to a passion for an effortful and necessary activity such as a person's work? Also, would we expect the same personality traits to explain either form of passion? So far, virtually no study has investigated the relationship between (a specific) passion and personality in the work context, although personality is a key topic in research on vocational/organizational behavior (Judge et al., 2002).

We assume that domain-specific work-passion is, at least in part, an expression of corresponding domain-specific personality features of a person. This assumption is based on findings of the person-job-fit research (Fouad, 2007) and research on vocational interests (Holland, 1997), both of which state that optimal job motivation results from a match between a person's characteristics and the characteristics of the job. The research on vocational interests describes vocational interests as stable person characteristics, comparable and correlated with personality traits. A person-job fit is given if the constellations, or profiles, of these interests match the combinations of requirements in a particular vocation (Holland, 1997).

## Hypotheses

In the following, we develop our specific hypotheses on the link between personality and entrepreneurial passion. Given that the five-factor model (Big Five approach) is the leading and best-validated trait approach to personality (John and Srivastava, 1999; McCrae and Costa, 2008), we examine the link between personality traits and passion by focusing on this Big Five level. One can distinguish between a variable-oriented vs. a person-oriented perspective when studying Big Five traits and thus the basic character of a person (Magnusson and Torestad, 1993; Asendorpf, 2003). Whereas, the variable-oriented perspective examines the isolated effects of the single Big Five traits, the person-oriented approach focuses on the role of the intra-individual constellation of the Big Five traits forming the unique, basic character of a person. We apply and compare both perspectives of conceptualizing personality traits in the present study. The variable-oriented perspective inspired us to examine the separate effects of the single Big Five traits as unspecific, broad personality features, and the person-oriented perspective was used to look at an entrepreneurial personality profile as a domain-specific personality feature (that has a clearer conceptual link to the specific work domain of entrepreneurship).

### Domain-Specific Personality (Entrepreneurial Personality Profile) vs. Broad Personality Dimensions (Single Big Five Traits) as Predictors of Entrepreneurial Passion

The person-oriented perspective tries to capture the entrepreneurial mindset of a person by studying the entrepreneurial constellations of traits within a person. Thus, an entrepreneurial personality profile (i.e., the entrepreneurial constellation of the Big Five traits) represents a domain-specific personality characteristic that is defined at a very basic, biologically related Big Five level and, at the same time, is conceptually linked to the target outcome—entrepreneurship. Such an entrepreneurial Big Five profile can be regarded as the relatively stable basic tendencies level in the entrepreneurial personality system (McCrae and Costa, 2008). Studies showed that such an intra-individual entrepreneurial Big Five profile (i.e., high in extraversion, conscientiousness, openness, low in agreeableness, neuroticism) is a robust predictor of a range of entrepreneurial outcomes such as entrepreneurial motivation, skills, networks, alertness, intentions, and behaviors (Schmitt-Rodermund, 2004; Obschonka et al., 2013, 2017; Obschonka and Stuetzer, 2017). This research delivered support for a system view on the entrepreneurial mindset (Obschonka and Stuetzer, 2017), according to which the entrepreneurial Big Five profile, as a basic entrepreneurial tendency that is substantially determined by the biological level, affects entrepreneurial behavior via characteristic adaptations. This perspective assumes that one such characteristic adaptation is entrepreneurial passion that develops out of a characteristic (following the basic, entrepreneurial character of a person) adaptation to the environment. This would thus also be in line with a general person-job fit approach (Holland, 1997). Hence, following this notion of a corresponsive principle where domain-specific personality features (e.g., entrepreneurial Big Five profile) should be particularly relevant for the development of corresponding domain-specific passion (e.g., entrepreneurial passion) in the entrepreneurial personality system, we expected to find an effect of the entrepreneurial Big Five profile (person-oriented approach) on entrepreneurial passion, whereas the unspecific, single Big Five dimensions (variable-oriented approach) should show no effect.

Hypothesis 1: An entrepreneurial personality profile (but not the single Big Five dimensions) positively predicts entrepreneurial passion.

### Mediation Effect of Passion

If (domain-specific) personality predicts corresponding (domain-specific) passion, then the latter should mediate the personality-behavior link. This follows from passion theories and research stressing that domain-specific work passion drives engagement (and persistence) in corresponding, domain-specific work behaviors/tasks due to the motivational effect of passion (see also Vallerand et al., 2003; Cardon et al., 2009b; Cardon and Kirk, 2015). As stressed in Cardon et al. (2013), entrepreneurial passion not only promotes “creativity and the recognition of new information patterns critical to the discovery and exploitation of promising opportunities,” but also facilitates social processes relevant in the entrepreneurial process (e.g., convincing investors or potential partners and employees) (p. 373).

Moreover, this mediation assumption also follows from the notion that domain-specific personality should be predictive of domain-specific behavior as it was, for example, demonstrated in the context of entrepreneurial behavior and the entrepreneurial Big Five profile (Obschonka and Stuetzer, 2017). In fact, the personality approach to entrepreneurship is widely regarded as the classical approach to the study of entrepreneurial activity (Hisrich et al., 2007), stressing that such activity could be (at least in part) an expression of a person's personality structure. The entrepreneurial personality profile might make entrepreneurial activity more likely because it stimulates the development of entrepreneurial characteristic adaptations (like entrepreneurial passion but also other malleable aspects of the entrepreneurial mindset like human and social capital and entrepreneurial cognitions) over the course of a person's vocational development across the life-span (Obschonka and Stuetzer, 2017).

Taken together we expected that the entrepreneurial personality profile (but not the single Big Five dimensions) affects entrepreneurial behavior via the mediating effect of entrepreneurial passion.

Hypothesis 2: Entrepreneurial passion mediates the link between an entrepreneurial personality profile and entrepreneurial behavior.

### Personality and Passionate Entrepreneurial Behavior

So far, we had assumed that passion should be studied as independent of behavior (e.g., as predictor and motivator of behavior). However, it is an empirical question whether or not entrepreneurial passion and entrepreneurial behavior occur together or independently. It seems plausible that some individuals may feel passionate about entrepreneurial activities, but lack the time, promising ideas, or skills to realize this passion in entrepreneurial behavior. Vice versa, could it be possible that some individuals show the entrepreneurial behavior and start a business without feeling a passion for it? We explored this question with cluster analysis and assumed that most individuals experience a coupling of entrepreneurial passion and behavior, in the sense that both are either low or high, within a person.

Continuing the assumption above that entrepreneurial passion and entrepreneurial behavior may often occur together within individuals, we next examined the effect of personality predictors on such coupled co-occurring passionate behavior. Such passionate entrepreneurial behavior might be a better fit to Vallerand's initial definition of passion for an activity that one is currently engaged in (one already shows the behavior in question and experiences passion while doing this behavior). Moreover, policymakers are particularly interested in understanding (drivers of) passionate entrepreneurship because an entrepreneur who is passionate about his or her entrepreneurial activity should be more productive, persistent, and happy with this activity than an entrepreneur without such passion (Cardon et al., 2009b). In fact, given that there are convincing arguments and research findings suggesting that entrepreneurial activity *without* entrepreneurial passion (“cold pathway”) can be seen as somewhat problematic as passion is at the heart of entrepreneurship, then entrepreneurs lacking such passion are likely to run into manifold problems, ranging from problems with self-motivation to social processes (e.g., convincing investors and potential employees, customers, and business partners, and maintaining the persistence, personal effort, and optimism during critical and challenging times in the entrepreneurial process) (Cardon et al., 2013). In other words, entrepreneurial behavior shown by individuals who also show a strong passion for this behavior should be a desired outcome in entrepreneurship policies (e.g., in the field of academic entrepreneurship) and should thus be explicitly addressed in empirical research. So we do not examine passion as an independent motivator of behavior in this part of the study but as an integrative part of behavior—the coupling of passion and behavior.

Following our earlier argumentation on the expectable importance of domain-specific personality for entrepreneurial passion, we assumed that the entrepreneurial Big Five profile should not only predict entrepreneurial passion, but also passionate entrepreneurial behavior where both actually come together, the activity and the passion in that domain (the combination that is of particular interest from a public policy perspective interested in academic entrepreneurship and passionate entrepreneurial behavior in academia, Shane, 2004). Again, we expected to find effects of the domain-specific personality profile but not for the unspecific, single Big Five dimension.

Hypothesis 3: The entrepreneurial Big Five profile (but not the single Big Five dimensions) positively predicts passionate entrepreneurial behavior.

## Methods

### Sample and Procedure

We analyze data from the Thuringian Founder Study (“Thüringer Gründer Studie”), an interdisciplinary German research project on the determinants of innovative entrepreneurship. One part of the project was a longitudinal online survey of scientists employed in German research institutions (e.g., universities, Max Planck Institutes, Fraunhofer Institutes) to examine entrepreneurial motivations in a population where the potential for innovative entrepreneurship (i.e., based on science-based business ideas) is particularly high. The data were collected along three waves. In June 2008 (T1), the main study was conducted which mainly targeted entrepreneurial intentions and its determinants, such as specific personality traits (for a detailed description of the T1 sample selection and data collection procedure see Goethner et al., 2012; Obschonka et al., 2012). Follow-up waves in December 2010 (T2) and in November 2012 (T3) assessed entrepreneurial behavior (at T2 and T3) as well as entrepreneurial passion (at T3). The procedure was evaluated as non-invasive, and APA's ethical principles and code of conduct were followed (ensuring privacy, anonymity, and confidentiality with respect to respondents' identity and data). An ethics approval was not required at the time the research was conducted as per our institution's guidelines (University of Jena, Germany) and national regulations. The consent of the participants was obtained by virtue of survey completion.

For this study, we used data on *n* = 137 respondents who participated in T1 and T3 of the data collection. The dataset is available under https://osf.io/wj5td/. The mean age of these respondents was 40.0 years (*SD* = 11.63, range: 23–65) and 65.7% were male. About two-thirds worked in a research university (61.3%), 11.9% worked in a university of applied sciences (“Fachhochschule”), and 25.2% worked in non-university research institutions. Regarding their occupational status, 75.2% of the participants worked as research associates, 16.8% were professors or university lecturers, and 8.0% reported another field of activity, for example, as a technical assistant. More than half of the sample (59.1%) described their type of engagement in research as applied science and the remainder (40.9%) as basic science. The largest group of participants worked in the field of natural sciences (53.7%), whereas 28.7% worked in engineering sciences and 17.6% in social sciences.

### Measures

In the following, we provide an overview of the measurement of the variables used in this study. Table 1 shows the means and standard deviations.

**Table 1:** Descriptives of study variables at baseline (T1) and attrition analysis (comparing T3 respondents and T3 dropouts).

	**T1**	**T3 response behavior**		**Difference respondents vs. dropouts**	
	**(*N* = 496)**	**Respondents (*n* = 137)**	**Dropouts (*n* = 359)**	***p*-value^a^**	**Effect size**
	***M (SD)***	***M (SD)***	***M (SD)***		
1. Entrepreneurial Personality Profile	−24.79 (6.68)	−24.26 (5.88)	−24.99 (6.96)	0.24	0.14
2. Extraversion	2.94 (0.66)	2.91 (0.68)	3.00 (0.59)	0.16	−0.17
3. Openness	3.23 (0.58)	3.24 (0.59)	3.23 (0.56)	0.90	0.01
4. Conscientiousness	3.39 (0.63)	3.38 (0.63)	3.42 (0.63)	0.54	−0.06
5. Neuroticism	1.68 (0.65)	1.71 (0.66)	1.61 (0.63)	0.15	0.13
6. Agreeableness	3.18 (0.56)	3.17 (0.55)	3.20 (0.59)	0.62	−0.04
7. Gender (1 = male, 0 = female)	0.70 (0.46)	0.66 (0.48)	0.72 (0.45)	0.16	−0.06
8. Age	38.79 (11.54)	40.01 (11.63)	38.32 (11.49)	0.15	0.13
9. Professor (1 = yes, 0 = no)	0.18 (0.39)	0.17 (0.38)	0.19 (0.39)	0.58	−0.03
10. Research (1 = applied; 0 = basic)	0.53 (0.50)	0.59 (0.49)	0.51 (0.50)	0.11	0.07
11. Expected benefits: Money	2.99 (1.18)	2.88 (1.20)	3.03 (1.17)	0.21	−0.11
12. Expected benefits: Reputation	2.81 (1.02)	2.78 (1.03)	2.82 (1.02)	0.71	−0.03
13. Expected benefits: Funding research	3.02 (1.10)	2.94 (1.09)	3.05 (1.10)	0.33	−0.09
14. Entrepreneurial Peers	1.34 (0.70)	1.32 (0.62)	1.34 (0.72)	0.77	−0.03
15. Group Identification	3.41 (0.78)	3.49 (0.77)	3.38 (0.78)	0.15	0.13

*Entrepreneurial behavior and entrepreneurial passion were only assessed at T3 and could therefore not be compared*.

a *Statistical significance of differences between respondents and dropouts was calculated using t-tests (Cohen's d) for continuous variables and χ^2^-tests (Cramer's V) for dichotomous variables*.

#### Harmonious Entrepreneurial Passion (T3)

In the T3 wave, we introduced items measuring passion into the questionnaire (this was not measured in the first two waves). Specifically, harmonious passion was measured with five items developed by Vallerand et al. (2003). Example items in this scale include “For me, being an entrepreneur is a passion” and “I am completely taken with being an entrepreneur” (five-point Likert scale). We estimated a latent factor to avoid measurement error in this outcome.

#### Entrepreneurial Behavior (T3)

Moreover, in the T3 wave, three items assessed respondents' entrepreneurial activity since the baseline in 2008 (“Since 2008: Have you been involved in consulting to companies to commercialize your research?”; “Since 2008: Did you apply for at least one patent to commercialize your research?”; “Since 2008: Have you participated in the founding of a new firm to commercialize your research?”; see Haeussler and Colyvas, 2011). Following Haeussler and Colyvas (2011), the three items were summed up for each respondent to create an index for the degree of engagement in entrepreneurship since 2008. Similar to Haeussler and Colyvas' (2011) study, the most frequent combination was consulting and patenting (14.8%).

#### Big Five Traits (T1)

We used a well-validated German 45-item questionnaire (Ostendorf, 1990) to derive the Big Five personality traits. This questionnaire was successfully employed in earlier entrepreneurship studies (e.g., Schmitt-Rodermund, 2004; Obschonka et al., 2011). *Extraversion* (e.g., “uncommunicative vs. talkative”), *conscientiousness* (e.g., “lazy vs. diligent”), *openness* (e.g., “conventional vs. inventive”), *agreeableness* (e.g., “good-natured vs. cranky”), and *neuroticism* (e.g., “vulnerable vs. robust”) were measured by nine six-point bipolar items each, with answers ranging from (0) to (5). Cronbach's α was ≥0.70 for each Big Five trait.

#### Entrepreneurial Personality Profile (T1)

To quantify an entrepreneurship-prone personality profile, we followed previous research (Obschonka et al., 2013) and applied a fit measure that summarizes the single Big Five traits into one index. This fit measure is comparable to Cronbach and Gleser's (1953) *D*^2^ profile similarity approach. By means of a fixed entrepreneurial reference profile with extreme scores as endpoints of the distributions (lowest possible score [0] in agreeableness and neuroticism; highest possible score [5] in extraversion, conscientiousness, and openness) the individual deviation from these statistical endpoints is assessed as *D*^2^. To do this, each respondent's squared differences between the reference values and their personal values on each of the Big Five scales were computed. For instance, if a respondent scored 3 in neuroticism, the squared difference was 9 (because the reference value was 0). The five squared differences were then summed up for each respondent, and the algebraic sign of this sum was reversed (e.g., a value of 20 became −20). The resulting index served as the final variable *entrepreneurship-prone personality profile*. The higher the score on this index (closer to zero), the stronger a respondent's entrepreneurial personality structure.

#### Control Variables (T1)

To test the personality approach against other major approaches to human motivation and organizational behavior, we considered the following alternative explanation models in the prediction of entrepreneurial passion and behavior.

##### Rational choice approach

A traditional economic approach to work motivation and outcomes is the rational choice approach, stressing the role of expected benefits a certain decision, role, or work outcome entails (Banerjee and Newman, 1993; Douglas and Shepherd, 2002). In the present case, these might be the potential, expected benefits an engagement in academic entrepreneurship could entail for the entrepreneurial scientist. In other words, a scientist who develops a passion for entrepreneurial work and engages in academic entrepreneurship may depend on rational estimations as to whether a personal engagement in academic entrepreneurship brings along more advantages and fewer disadvantages than other occupational options. Research has found, for example, that expected benefits associated with an engagement in academic entrepreneurship (e.g., expected reputational gain due to additional scientific reputation given the commercialization of own research findings via academic entrepreneurship; higher personal income due to the additional entrepreneurial income) predict a positive attitude and self-efficacy beliefs with regard to own academic entrepreneurship in scientists (Goethner et al., 2012). For this perspective, one could thus argue that scientists “fall in love with” and thus engage in academic entrepreneurship as a consequence of expected benefits compared to non-entrepreneurial work. If academic entrepreneurship entails more perceived benefits and incentives than other occupational options (e.g., a pure focus on regular scientific work in the ivory tower), the scientist might be more motivated and passionate to act entrepreneurially.

We thus included three control variables capturing potential benefits. The items were preceded by the stem “Please assess the likelihood of these consequences if you were to participate in the founding of a firm in order to commercialize your own research.” The first consequence referred to expected reputational gain and was measured with the item “Additional scientific reputation” (1 = very unlikely, 5 = very likely). The second consequence referred to expected financial gain and was measured with the item “Higher personal income” (1 = very unlikely; 5 = very likely). The third item referred to the topic of funding own research and was measured with “Additional funds to support own research” (1 = very unlikely, 5 = very likely).

##### Social learning approach

Beside personality and expected benefits, social learning approaches have been proven to be another particularly useful perspective in work and vocational psychology (Lent et al., 1994; Fouad, 2007) as well as in the specific field of entrepreneurship research (Chen et al., 1998; Baum and Locke, 2004). This directs the attention toward the social context in which vocational behavior and development are embedded (Vondracek et al., 1986; Silbereisen, 2002). Based on seminal works on social learning and socio-cognitive theories giving (learning from) role models a unique role in motivational processes (e.g., Bandura, 1982), for example in vocational behavior and performance (Lent et al., 1994), such social learning processes can be powerful shapers of individual motivation and behavior (Gibson, 2004), particularly with respect to challenging tasks (such as entrepreneurship). One can thus expect that having entrepreneurial role models (entrepreneurial workplace peers) makes it more likely that the individual scientist engages in own passionate entrepreneurial behavior (Nanda and Sørensen, 2010). Role models should stimulate entrepreneurial self-efficacy beliefs, which in turn might foster passion (Cardon and Kirk, 2015), but existing research is not univocal and clear about a causal effect of self-efficacy on passion. Moreover, if a scientist works together with entrepreneurial workplace peers there might be a certain collective passion for entrepreneurship present “in the lab,” which in turn might foster entrepreneurial passion in the individual scientist him/herself (Cardon, 2008). We, therefore, included entrepreneurial workplace peers as a control variable. We measured *entrepreneurial workplace peers* using a three-item scale assessing entrepreneurial behavior in respondents' superiors, colleagues, and co-authors. Respondents were asked: “Please estimate how many persons in your work environment have already engaged in academic entrepreneurship?” (item 1: “among your superiors”; item 2: “among your workplace colleagues”; item 3: “among your coauthors”) (five-point Likert scale; 1 = nobody, 5 = everybody; α = 0.66).

#### Social Identity Approach

While the social learning perspective already directs attention toward the organizational context, workplace peers, another important context-related factor might be if the individual scientist actually identifies him or herself with these workplace peers. This refers to the social identity of individuals (e.g., group identification), which has been another major research focus in research on work and vocational psychology across the past decades (e.g., Gottfredson, 1981; Ashforth and Mael, 1989; Van Knippenberg, 2000). Identity issues have also been receiving increasing interest in contemporary entrepreneurship research (Fauchart and Gruber, 2011; Miller and Breton-Miller, 2011). Drawing from a social identity perspective (Stets and Burke, 2000), it can be expected that entrepreneurial workplace peers only affect entrepreneurial motivation when the individual scientist identifies him or herself with these workplace peers (Terry and Hogg, 1996; Obschonka et al., 2012). We thus tested *group identification with workplace peers* as a predictor and also as a moderator of the workplace peers and passion link. Three items assessed respondents' identification with their academic workplace peers (Terry and Hogg, 1996) (Item 1: “Generally speaking, how much do you identify with your group of colleagues at the university/research institute?”; Item 2: “Personally, how strong is your sense of belonging to the group of your colleagues at the university/research institute?”; Item 3: “Do you share social bonds with your colleagues at the university/research institute?”); (five-point Likert scale; 1 = not at all, 5 = totally; α = 0.77).

Finally, further socio-demographic standard control variables were added. As the level of entrepreneurial behavior may vary with an individual's background and life experience (Cardon et al., 2013), we include *age* (measured in years) and *gender* (0 = female, 1 = male) as control variables. We also control for academic seniority effects. Several authors have found a positive relationship between academic rank and scientists' commitment toward entrepreneurial efforts (see Perkmann et al., 2013). As a proxy for academic rank, the dummy variable *professor* takes the value 1 if the respondent is a professor or university lecturer and 0 otherwise. Finally, we control for the type of *research* (0 = basic vs. 1 = applied).

### Statistical Method

To test our hypotheses, we employed structural equation modeling (SEM) in Mplus (version 7.31; Muthén and Muthén, 2008–2015). Missing data were handled with Full Information Maximum Likelihood Estimation (FIML; Enders and Bandalos, 2001). This method does not impute or replace missing data but estimates values for missing variables based on all variables included in the estimated model.

### Attrition Analysis

It is important to note that the follow-up waves in T2 and T3 suffer from considerable sample attrition of 58.7 and 40.5%, respectively. Hence, the T3 sample (*n* = 137) used to test our hypotheses represents only about a quarter (24.6%) of the T1 baseline sample (*n* = 496). This attrition rate raises the issue of whether our results may be biased because of non-random and selective inclusion in the final T3 sample. To assess the presence of potential attrition bias, we compared respondents and dropouts in each wave on all study variables measured at baseline (Menard, 2002).

For dichotomous variables, differences between both groups were tested with the χ^2^-test of independence and the effect size Cramer's V. For continuous measures, mean differences were tested with the *t*-test for independent samples, and effect size Cohen's *d*. Statistical tests were considered significant at the *p* ≤ 0.05 level. Additionally, Cohen's *d* statistics were computed to assess the effect size of differences between respondents and dropouts regarding the distribution of the study variables. Cohen's (1988) conventions for evaluating effect sizes were applied, where a *d* of 0.20 is regarded as a weak or small association; a *d* of 0.50 is considered a moderate effect; and a *d* of 0.80 or larger represents a strong or large effect.

As reported in Table 1, χ^2^- and *t*-tests showed no significant differences (at *p* ≤ 0.05) between participants who took part in the T3 follow-up and those who did not in any of the main or control variables measured at T1. Thus, sample attrition does not seem to affect the variance of the key measures in our study. This allows us to conclude that sample attrition at T3 should not substantially affect the pattern of results derived from our analysis.

## Results

### Zero-Order Bivariate Correlations

Table 2 presents the zero-order correlations between the variables. Entrepreneurial passion was correlated with being male, and with doing applied research (0.18 ≤ *r* ≤ 0.30). Entrepreneurial behavior correlated moreover with being male, age, being a professor, applied research, and having entrepreneurial peers (0.18 ≤ *r* ≤ 0.34). Having entrepreneurial peers was correlated positively with being male, being older, being a professor, doing applied research and expecting monetary benefits. Group identification was correlated only with the entrepreneurial personality profile.

**Table 2:** Correlations of the Variables.

	**2**	**3**	**4**	**5**	**6**	**7**	**8**	**9**	**10**	**11**	**12**	**13**	**14**	**15**	**16**	**17**
1. Entrepreneurial Personality Profile	0.66^**^	0.53^**^	0.48^**^	−0.59^**^	−0.33^**^	−0.02	0.09^*^	0.12^**^	0.03	0.16^**^	0.10^*^	0.13^**^	0.07	0.16^**^	0.19^*^	0.18^*^
2. Extraversion	1	0.34^**^	0.18^**^	−0.38^**^	0.06	−0.10^*^	0.10^*^	0.08	0.06	0.14^**^	0.19^**^	0.12^*^	0.09^*^	0.04	0.08	0.13
3. Openness		1	0.16^**^	−0.24^**^	0.05	−0.03	0.04	0.14^**^	−0.03	0.16^**^	0.05	0.12^**^	0.14^**^	0.13	0.25^**^	0.15
4. Conscientiousness			1	−0.33^**^	0.18^**^	−0.05	0.11^*^	0.06	0.04	0.01	0.11^*^	0.08	−0.00	0.12	0.13	0.09
5. Neuroticism				1	−0.20^**^	−0.16^**^	−0.14^**^	−0.16^**^	−0.08	−0.14^**^	−0.20^*^	−0.15^**^	−0.11^*^	−0.18^*^	−0.27^**^	−0.26^**^
6. Agreeableness					1	0.01	0.12^**^	0.05	0.04	−0.04	0.09^*^	0.05	0.06	0.06	0.08	0.04
7. Gender (1 = male)						1	0.23^**^	0.25^**^	0.13^**^	0.05	−0.10^*^	−0.01	0.21^**^	0.01	0.18^*^	0.29^**^
8. Age							1	0.50^**^	0.15^**^	−0.12^**^	0.08	0.07	0.16^**^	−0.05	−0.00	0.34^**^
9. Professor (1 = yes, 0 = no)								1	0.03	0.02	0.04	0.06	0.10^*^	−0.09	0.02	0.24^**^
10. Research (1 = applied; 0 = basic)									1	0.12^**^	0.13^**^	0.11^*^	0.14^**^	−0.02	0.30^**^	0.30^**^
11. Expected benefits: Money										1	0.17^**^	0.40^**^	0.11^*^	0.01	0.12	−0.01
12. Expected benefits: Reputation											1	0.40^**^	0.04	0.05	0.14	0.12
13. Expected benefits: Funding research												1	0.07	0.01	0.15	0.12
14. Entrepreneurial Peers													1	−0.01	0.06	0.20^*^
15. Group Identification														1	−0.05	0.01
16. Entrepreneurial Passion															1	0.40^**^
17. Entrepreneurial Behavior																1

^***^p < 0.001,

** p < 0.01,

* *p < 0.05, n of observations = 137*.

The entrepreneurial personality profile correlated with the covariates age, being a professor, expected benefits regarding money, reputation and research funding, and group identification. The entrepreneurial personality profile also correlated significantly with the outcomes (*r*_*entrepren*.*passion*_ = 0.19^*^, *r*_*entrepren*.*behavior*_ = 0.18^*^). Among the Big Five personality factors, openness was significantly correlated with entrepreneurial passion (*r* = 0.25^**^), but not with entrepreneurial behavior. Neuroticism was negatively correlated with both entrepreneurial passion (*r* = −0.27^**^) and entrepreneurial behavior (*r* = −0.26^**^). All other Big Five factors were unrelated to entrepreneurial passion and behavior.

### Main Results

#### Results for the Models With Passion and Behavior as Separate Constructs

**Figure 1:**
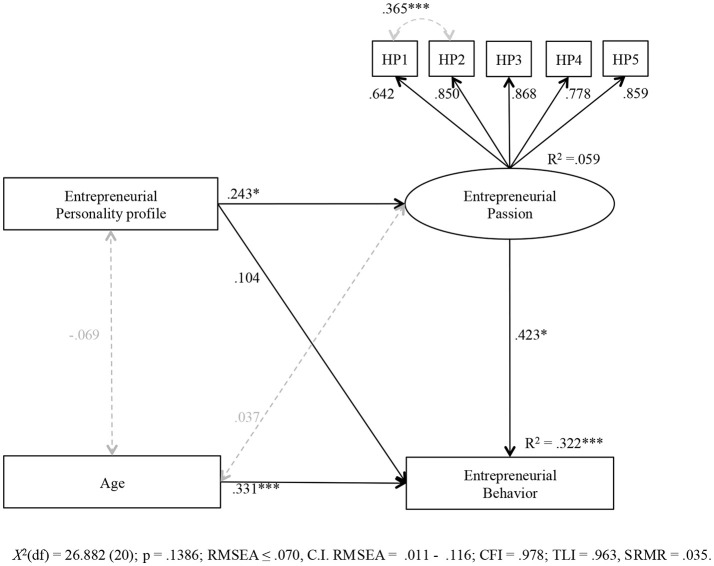
Mediation model with the entrepreneurial personality profile as independent variable. Standardized coefficients are given. Dashed lines represent correlations. *p < 0.05, **p < 0.01, ***p < 0.001. A previous model included all covariates (gender, age, being a professor, and doing applied research; expecting monetary benefits, expecting benefits for reputation, expecting benefits for research funding, entrepreneurial peers, and identification with peers), but for this final model we kept only the covariate with significant effect (age).

We then examined whether the entrepreneurial personality profile, vs. the single Big Five dimension, predicts entrepreneurial passion (Hypothesis 1), and whether passion mediates the link between personality and behavior (Hypothesis 2). In a first step, we considered all control variables (socio-demographics, including gender, age, being a professor, and doing applied research; and alternative explanations for passion/behavior: rational choice approach, social learning approach, and social identity approach). Then, for the final model, we left out the non-significant control variables to report a parsimonious model. The final model thus only includes age as a relevant control variable (because it showed a positive effect on behavior)—all other control variables turned out as irrelevant. The empirical SEM model with the entrepreneurial personality profile as a predictor (controlling for age) is shown in Figure 1. Supporting Hypothesis 1, the profile positively predicted passion (β = 0.24^*^). The variance of passion explained in this model was *R*^2^ = 0.06. In contrast, the empirical SEM model with the single Big Five dimensions (controlling for age) did not deliver any significant effects of the Big Five on passion (Figure A1 in Supplementary Material). The standardized regression coefficients for the Big Five traits ranged between −0.20 (for neuroticism) and 0.19 (for openness).

We then tested the mediating effect of passion between personality and behavior (estimating indirect effects and their bootstrap 95% confidence interval). The results are summarized in Table 3. For the entrepreneurial personality profile, there was a significant mediation effect as the 95% confidence interval of the indirect effect (β = 0.10) did not include zero. For the single Big Five, all confidence intervals included zero. Hence, Hypothesis 2 also received support: The entrepreneurial personality profile, but not the single Big Five traits, affected entrepreneurial behavior via increased entrepreneurial passion.

**Table 3:** Mediation tests for the entrepreneurial personality profile (Figure 1) and the Big Five traits (Figure A1 in Supplementary Material).

	**Total effect (standardized)**	**Specific indirect effect (standardized)**	**95% confidence interval**
**MODEL 1:**			
Entrepreneurial personality profile	0.207**	0.103	0.001 to 0.031
**MODEL 2:**			
Conscientiousness	0.055	0.039	−0.069 to 0.175
Extraversion	0.046	0.012	−0.099 to 0.154
Agreeableness	−0.087	−0.009	−0.135 to 0.091
Openness	0.081	0.076	−0.013 to 0.242
Neuroticism	−0.197*	−0.083	−0.240 to 0.006

#### Passionate Entrepreneurial Behavior (Coupling of Passion and Behavior)

To complement our analysis, we then turned to the question of the coupling of passion and behavior, as deemed particularly central in passion theory (Vallerand et al., 2003), approaches to entrepreneurship (Cardon et al., 2009b), and public policy (e.g., targeting and promoting passionate entrepreneurs where individuals engaged in entrepreneurial activity also show high passion for this activity). As shown in Table 2, we saw a substantial zero-order correlation between entrepreneurial passion and entrepreneurial behavior (*r* = 0.40^**^). This correlation indicates a considerable overlap between passion and behavior (e.g., “passionate behavior”). However, even this substantial correlation does not automatically imply that entrepreneurial passion and behavior co-occur in all individuals. Since based on previous findings one can expect that some individuals might experience entrepreneurial behavior without passion (“cold pathway”) or vice versa, we examined the intra-individual profiles of passion and behavior. For that purpose, we conducted a two-step cluster analysis with passion and behavior as the two indicators (using log-likelihood distance measure and Schwarz's Bayesian Clustering Criterion; BIC). Four clusters resulted, and the average silhouette measure of cohesion and separation was good (0.6). To facilitate the interpretation of the clusters, we transformed the response scales of the two indicators passion and behavior so that they were brought to the same scale, ranging from 0 to 1, using the Proportion of Maximum Scaling Transformation (POMS; Little, 2013). The first cluster displayed very low levels of entrepreneurial passion and behavior and comprised 25.5% of individuals in our sample. The second cluster displayed moderate levels of entrepreneurial passion and behavior, comprising 23.4% of the individuals. The second cluster is thus clearly in line with the coupling perspective. Interestingly, the third cluster displayed high levels of passion but low levels of entrepreneurial behavior. However, this cluster was rather small (14.6% of all individuals) and one could argue that it reflects the group of scientists who already showed the passion, however had no chance to engage in own entrepreneurial activity yet, but might do so in the near future (so that the coupling of passion and behavior might eventually be achieved). The fourth and largest cluster showed high levels of entrepreneurial passion and behavior and thus represents the “hot pathway” of passionate behavior (36.5% of all individuals). Taken together, we found relatively strong empirical support for the actual coupling of passion and behavior. Noteworthy, we did not observe any cluster resembling the “cold pathway” of entrepreneurial behavior without entrepreneurial passion.

After establishing this coupling of passion and behavior empirically, we tested an SEM model with a latent factor for “passionate entrepreneurial behavior” including the indicators of entrepreneurial passion and behavior. Both indicators loaded strongly on this factor (*r*_*behavior*_ = 0.70 and *r*_*harmonious*_*passion*_ = 0.65; see **Figure 3**). This latent factor represents the coupled experience of passionate entrepreneurial behavior that we had identified as the dominating pattern (either low/low, moderate/moderate, or high/high) in the cluster analysis.

Then we examined the effect of personality on this latent factor of passionate entrepreneurial behavior. As covariates, we again included only those control variables that showed a significant effect, in this case, gender and type of research. All other control variables (e.g., the alternative explanations) again turned out as irrelevant. The results for the effect of the entrepreneurial personality profile are shown in **Figure 3**. The entrepreneurial personality profile had a significant positive effect on passionate entrepreneurial behavior (β = 0.27^**^), which supports Hypothesis 3. Moreover, males were more likely to report higher levels of passionate entrepreneurial behavior than females (β = 0.29^**^), and applied researchers were more likely to report higher levels of passionate entrepreneurial behavior than basic researchers (β = 0.36^*^). The variance of passionate entrepreneurial behavior explained by these predictors was substantial (*R*^2^ = 0.35).

Finally, Figure A2 in Supplementary Material shows the SEM model including the single Big Five traits instead of the entrepreneurial profile. It revealed that none of the Big Five personality factors was a significant predictor of passionate entrepreneurial behavior, in contrast to the entrepreneurial personality profile. The standardized regression coefficients for the Big Five traits ranged between −0.20 (for neuroticism) and 0.16 (for openness).

## Discussion

The current study investigated the effects of personality traits and personality structure on entrepreneurial passion and entrepreneurial behavior in organizations, using the example of entrepreneurial passion and behavior in academia (academic entrepreneurship). We compared the effects of domain-specific personality features vs. those of broad, unspecific personality traits on passion and behavior. Second, we also took a closer look at the motivating role of passion (studied independently as a mediator between personality and behavior) vs. the coupling of passion and behavior when studied as two indicators of the latent construct–passionate entrepreneurial behavior. Our results deliver four central messages.

**Figure 2:**
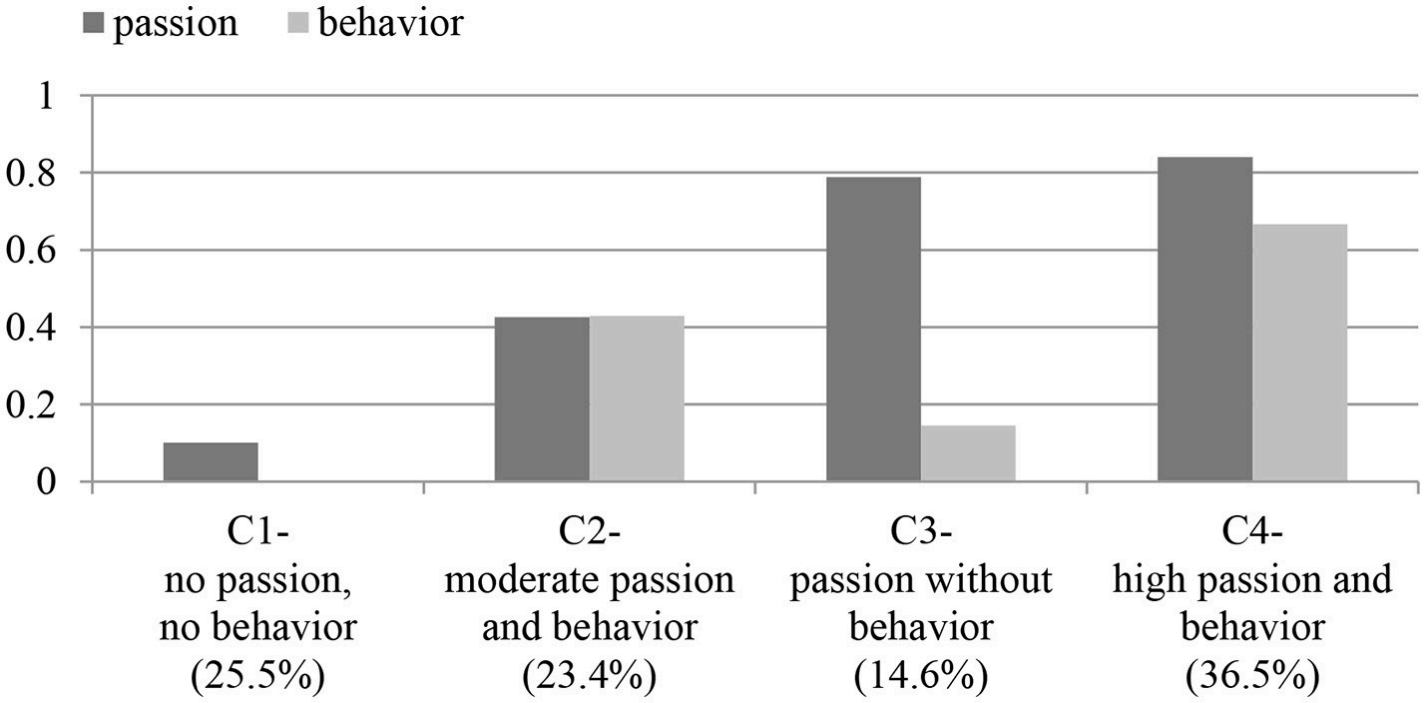
The coupling of entrepreneurial passion and behavior: Four clusters in the two-step cluster analysis.

**Figure 3:**
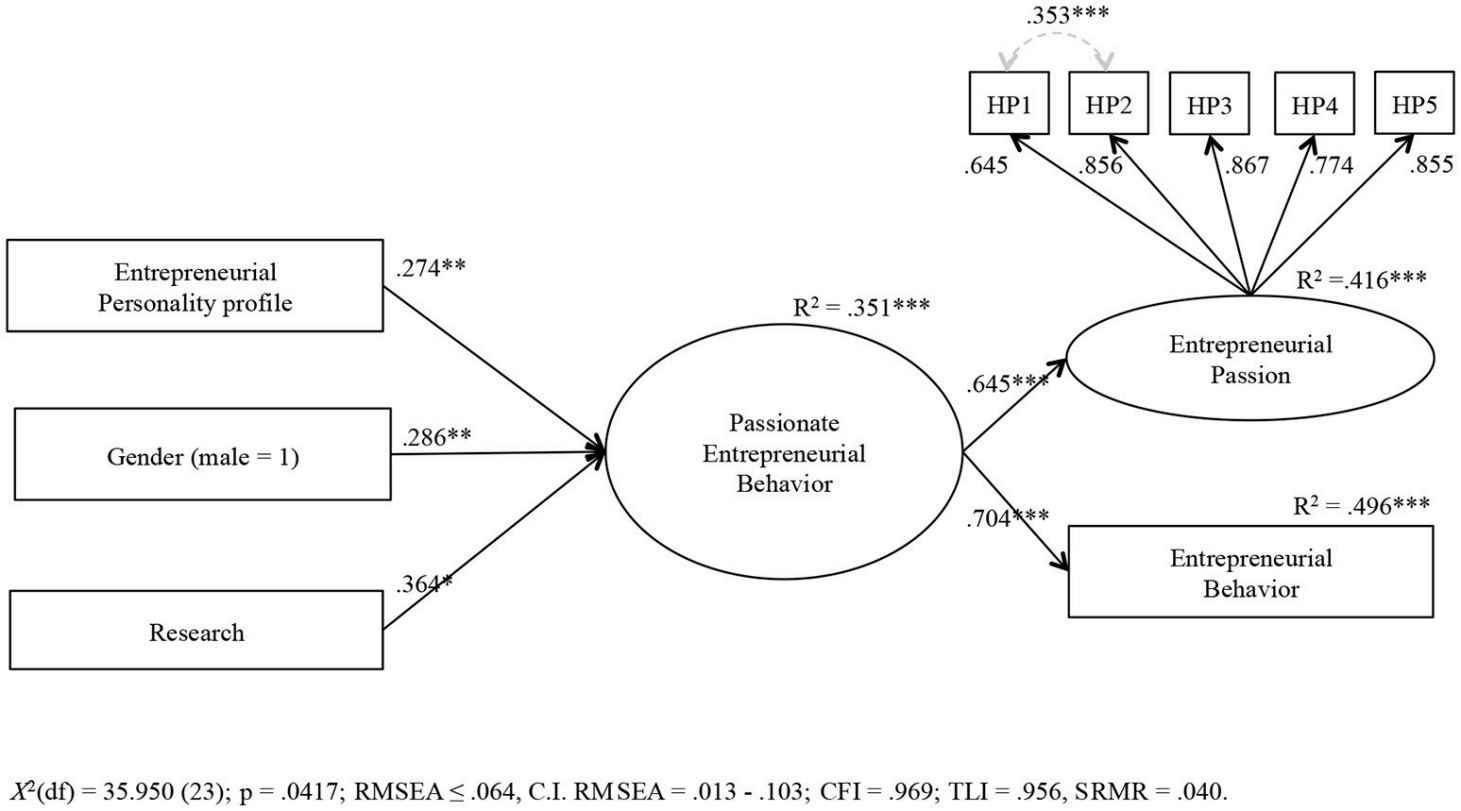
Effect of the entrepreneurial personality profile on passionate entrepreneurial behavior. Standardized coefficients are given. Correlations between the predictors lead to model misspecifications and were therefore not included in the model. Only gender and research were correlated (*r* = 0.209). *p < 0.05, **p < 0.01, ***p < 0.001. A previous model included all covariates (gender, age, being a professor, and doing applied research; expecting monetary benefits, expecting benefits for reputation, expecting benefits for research funding, entrepreneurial peers, and identification with peers), but for this final model we kept only the covariate with significant effect (gender and doing applied research).

First, we contribute to entrepreneurship research by shedding light on the antecedents of entrepreneurial passion. We found that the domain-specific personality feature (the entrepreneurial trait profile) predicted entrepreneurial passion (Figure 2) and passionate entrepreneurial behavior (Figure 3). By contrast, the domain-unspecific single Big Five traits had no significant effects (Figures A1, A2 in Supplementary Material). This underscores the assumption that a basic entrepreneurial character of a person, as, for example, conceptualized by the entrepreneurial constellation of the Big Five traits as the basic tendencies level in the personality system (McCrae and Costa, 2008; Obschonka and Stuetzer, 2017), gets expressed via characteristic adaptations (e.g., entrepreneurial passion) if the right conditions are present in an organization (in our case new research findings that can serve the basis for own entrepreneurial activities of research scientists, Shane, 2004, and the positive and stimulating atmosphere toward academic entrepreneurship in today's academic institutions, Perkmann et al., 2013; Huyghe et al., 2016). One channel through which such a personality feature might affect such a passion could be identity formation processes that are known to play a central role in the development of passion (Vallerand et al., 2003; Cardon et al., 2009b; Murnieks et al., 2014). As an important developmental dynamic in the entrepreneurial personality system (Obschonka and Stuetzer, 2017), the basic entrepreneurial character guides identity formation in a person's vocational development (see also McCrae and Costa, 2008). Indeed, research suggests that the entrepreneurial personality profile leads to higher levels of entrepreneurial self-identity (Obschonka et al., 2015).

Second, our analyses suggest that entrepreneurial passion and behavior tend to couple (co-occur within individuals) in organizations. This informs entrepreneurship research interested in the question whether passion and activity can be studied separately or as a unit (Huyghe et al., 2016). While we found individuals who reported passion without the corresponding behavior, we did not find any evidence for a “cold pathway” (entrepreneurial behavior without passion) in our cluster analysis. In other words, the results underline a “positive” pattern in the real world where entrepreneurial behavior is indeed coupled with passion (e.g., both are high), which would be good news for public policy focusing on passionate entrepreneurial behavior because this should be the ideal pattern from a motivational perspective.

Third, our study also contributes to research on the “entrepreneurial scientist” (Aldridge and Audretsch, 2011). While our data cannot deliver any conclusion on entrepreneurial passion in other types of organizations besides academia, our results seem to suggest that the “entrepreneurial scientist” exists who indeed is often also passionate about entrepreneurship. Nevertheless, many scientists *do not* engage in own entrepreneurial activity at all and do not show the respective passion, even if their research has significant commercial potential (such as in Engineering Science and Life Sciences), and external conditions are stimulating (e.g., due to a promotive atmosphere in research institutions and concrete promotion programs offered by technology transfer and spinoff centers). One explanation for this could be (relatively stable) inter-individual differences in the basic trait character of scientists, as suggested in the current study. In fact, there is a substantial share of research-active scientists who score relatively low in the entrepreneurial personality profile, which could help explain why entrepreneurship in academia is (still) relatively scarce. This also fits with Holland's (1997) hexagon model of vocational interests according to which scientists are more of an investigative, intellectual type, and less of an enterprising doer type. So it might make sense to accept, to a certain degree, that many scientists might not develop entrepreneurial passion and a strong motivation to engage in own (passionate) entrepreneurial activity to commercialize their new research knowledge because it does not fit their basic character (and thus also their self-identity/occupational self-concept). Achieving a good fit between one's basic character and the nature of the job can be regarded as a central path toward a successful, satisfactory, and stable career (see Fouad, 2007). However, one could, of course, also argue that non-entrepreneurial scientists could team up with more entrepreneurially-minded team founders (i.e., “surrogate entrepreneurs,” Franklin et al., 2001) to start an academic spinoff that is based on their research findings. While these scientists might still not develop a strong passion for such entrepreneurial projects, the necessary passion could come from their founding partners.

Fourth, we also considered alternative explanations for human motivation and organizational behavior, namely the rational choice approach (expected benefits compared to non-entrepreneurial work), the social learning approach (role models in academic peers), and the social identity approach (personal identification with role models). Interestingly, none of these alternative explanations contributed to the explanation of passion and behavior in our study. This suggests that such a crucial and far-reaching personal decision like engaging in own entrepreneurial work (when started within an organizational setting) is not merely a result of external stimuli (e.g., expected benefits or entrepreneurial peers with whom one identifies). Instead, such a personal decision seems to be more the result of a good fit between one's basic entrepreneurial character and the opportunities (e.g., new research findings and stimulating environment). People differ in their basic trait structure and thus show a different propensity (and probably also willingness) to engage in passionate work behaviors of different kinds. However, it seems that if people achieve a good fit between their basic trait structure and characteristics and tasks of the job then work passion for the specific tasks seems to be more likely, given that passion is indeed, at least in part, an expression of this trait structure. However, we have to stress that other drivers of passion might also play a role, drivers that we did not study in our project. Indeed, substantial shares of the variance in entrepreneurial passion and passionate entrepreneurial behavior remain unexplained.

Our study has several limitations. First, our dataset does not include pre-post measures of passion and related behavior, which limits our implications concerning the change over time in these outcomes. Future research could determine how strongly such specific work passion changes and fluctuates—our results would suggest that it is fairly stable (given the underlying effect of the relatively stable trait character).

Second, we only examined harmonious passion. Future research could also look at obsessive passion and other components of entrepreneurial passion (e.g., for overviews, please see Cardon et al., 2009b, 2013; Moeller, 2014), including general (domain-unspecific) work passion and other domain-specific work passions (e.g., scientific work passion in scientists, Huyghe et al., 2016). Interesting new measures to capture entrepreneurial passion have been developed by Cardon et al. (2013). While these measures had not been available by the time our study was conducted, they may help future studies to understand the many components of the multi-faceted construct of entrepreneurial passion. Since passion is a multifaceted construct, it is important to keep in mind that our findings only refer to the components we assessed and that more studies are needed in order to find out whether the links between passion and the entrepreneurial personality profiles replicate if other components of passion are included.

Third, our sample was relatively small and only allowed us to study entrepreneurial motivation among academics, but not among individuals working in other types of organizations with similar entrepreneurial potential, like scientists working in corporate R&D departments. Replication studies are therefore needed. Finally, we did not directly compare the effect of the profile vs. the single Big Five dimensions in the same model, since there are several concerns regarding the validity of such an empirical head-to-head test of person- vs. variable-oriented approaches (Asendorpf, 2003).

Fourth, future research could test the generalizability of our results by employing other types of personality questionnaires and data collection method (e.g., estimating personality traits by means of computerized text analysis, Obschonka and Fisch, 2018, peer reports, McCrae and Costa, 1987, or behavioral analyses, Gosling et al., 2002).

Finally, future research could test whether our results also apply in other cultures. For example, studies could test which personality traits and profiles underlie entrepreneurial passion in non-Western cultures (Obschonka et al., 2018).

To conclude, our results introduce the basic entrepreneurial character, conceptualized at the personality traits level (McCrae and Costa, 2008), as a driver of entrepreneurial passion. While other, unobserved drivers also might play a role, it seems that we cannot have a complete understanding of entrepreneurial passion if we disregard the basic entrepreneurial personality character of a person.

## Ethics Statement

The present study is part of the interdisciplinary research project Thuringian Founder Study (Thüringer Gründer Studie). This large-scale project examines the process of business foundation in the Federal State of Thuringia, Germany, from the perspective of economics and psychology. In this paper, we present data from an online survey collected in 2008 (2010 and 2012). We followed APA's ethical principles and code of conduct and ensured privacy, anonymity, and confidentiality with respect to study subjects identity and results, and the analyses of the data in general. The nature of the data is non-invasive and participation in the study was voluntary (including the follow-up surveys) and we informed participants that we process their information as anonymized data. Given such an uncritical study design, ethical clearance was not necessary in 2008. This datasets has been used in severeal publications (examining other topics) published in journals such as Journal of Economic Psychology and Journal of Vocational Behavior (http://www.apa.org/ethics/code/).

## Author Contributions

MO planned the study, collected the data, and wrote the paper. JM analyzed the data and wrote the paper. MG collected the data and wrote the paper.

### Conflict of Interest Statement

The authors declare that the research was conducted in the absence of any commercial or financial relationships that could be construed as a potential conflict of interest.
