# Supplementary material for: Entrepreneurial Passion and Personality: The Case of Academic Entrepreneurship
Source: Front Psychol. 2019 Jan 9;9:2697. doi: 10.3389/fpsyg.2018.02697 (PMC6335975; doi:10.3389/fpsyg.2018.02697)
Supplement: Supplementary file 1 [file Table_1.DOCX]

APPENDIX

*Figure A1:* Mediation model with the single Big Five traits as independent variables. *Note.* Standardized coefficients are given. Dashed lines represent correlations. **p* < .05. ***p* < .01. ****p* < .001. Correlations among predictors were included in the estimated model. A previous model included all covariates (gender, age, being a professor, and doing applied research; expecting monetary benefits, expecting benefits for reputation, expecting benefits for research funding, entrepreneurial peers, and identification with peers), but for this final model we kept only the covariate with significant effect (age).
