# Supplementary material for: Entrepreneurial Passion and Personality: The Case of Academic Entrepreneurship
Source: Front Psychol. 2019 Jan 9;9:2697. doi: 10.3389/fpsyg.2018.02697 (PMC6335975; doi:10.3389/fpsyg.2018.02697)
Supplement: Supplementary file 2 [file Table_2.DOCX]

APPENDIX

**

*Figure A2:* Effect of the single Big Five trait on passionate entrepreneurial behavior. *Note.* Standardized coefficients are given. **p* < .05. ***p* < .01. ****p* < .001. Correlations among predictors were included in the estimated model. A previous model included all covariates (gender, age, being a professor, and doing applied research; expecting monetary benefits, expecting benefits for reputation, expecting benefits for research funding, entrepreneurial peers, and identification with peers), but for this final model we kept only the covariate with significant effect (gender and doing applied research).
